# Supplementary material for: Universal Constraints on Protein Evolution in the Long-Term Evolution Experiment with Escherichia coli
Source: Genome Biol Evol. 2021 Apr 15;13(6):evab070. doi: 10.1093/gbe/evab070 (PMC8233687; doi:10.1093/gbe/evab070)

SUPPLEMENTARY MATERIAL

Supplementary Figure S1. The density of observed mutations per gene across all hypermutator LTEE populations anti-correlates with mRNA and protein abundance at all time points, when genes with no mutations are excluded. RNA and protein abundance were measured for the ancestral LTEE clone REL606, grown in DM500 media (Caglar et al. 2017). Each point represents a protein-coding gene in the genome of the *E. coli* B strain REL606. The abundance of mRNA or protein expressed per gene is shown on the x-axis of each plot. The density of observed mutations per gene is shown on the y-axis of each plot. Comparisons to mRNA abundance are shown in purple, while comparisons to protein abundance are shown in green. Statistically significant linear regressions are shown in blue, while non-significant regressions are shown in light gray.

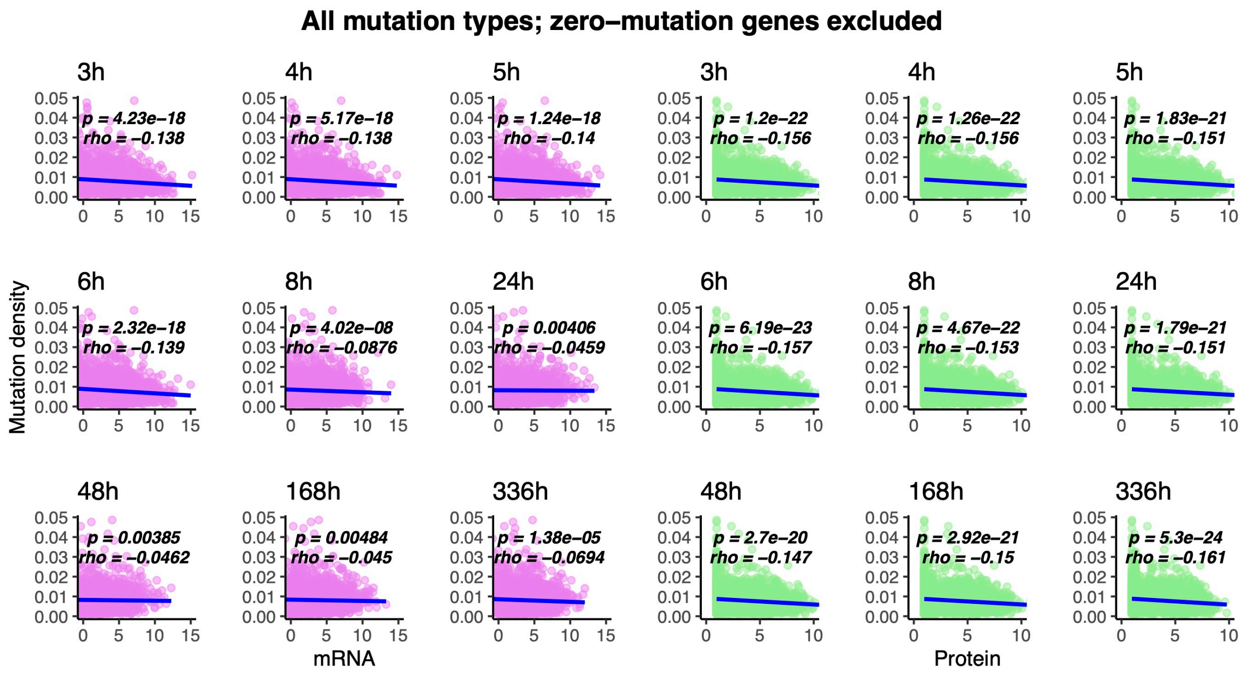

Supplementary Figure S2. The density of observed nonsynonymous mutations per gene across all hypermutator LTEE populations anti-correlates with mRNA and protein abundance at all time points, when genes with no mutations are excluded. See legend to Supplementary Figure S1 for further details.

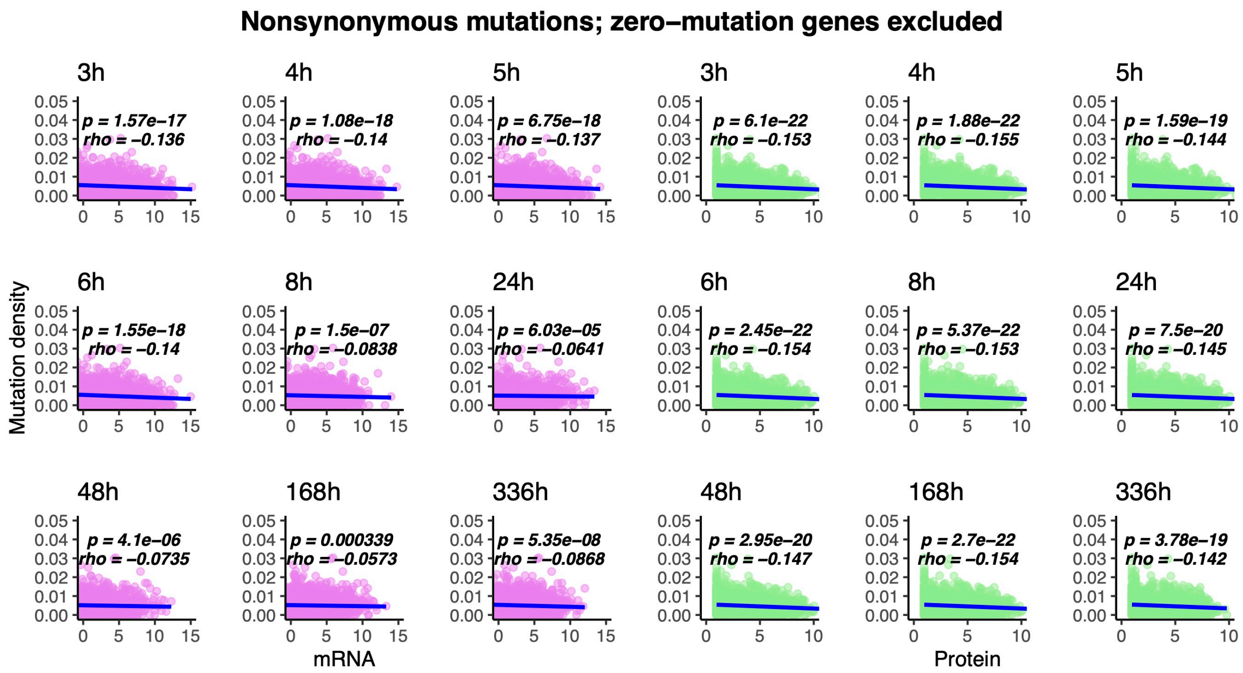

Supplementary Figure S3. The density of observed synonymous mutations per gene across all hypermutator LTEE populations positively correlates with mRNA abundance in exponential growth phase, and positively correlates with protein abundance at all time points, when genes with no mutations are excluded. See legend to Supplementary Figure S1 for further details.

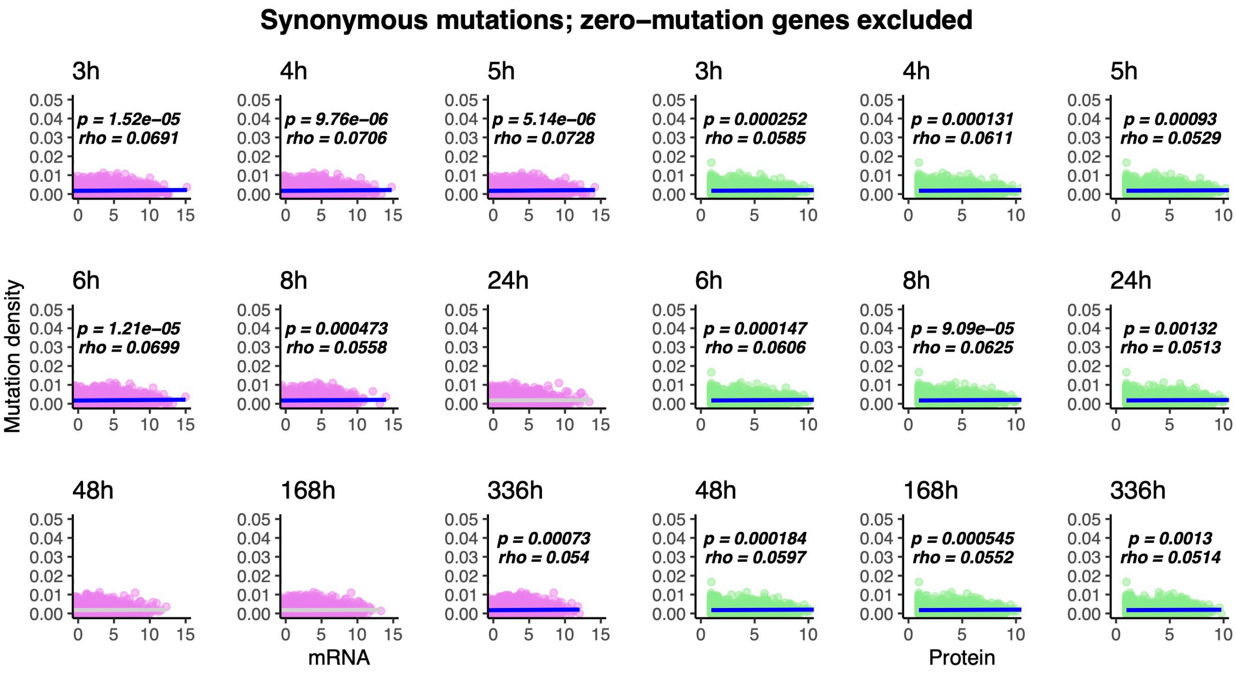

Supplementary Figure S4. The density of observed mutations per gene across all nonmutator LTEE populations positively correlates with mRNA and protein abundance at all time points. See legend to Supplementary Figure S1 for further details.

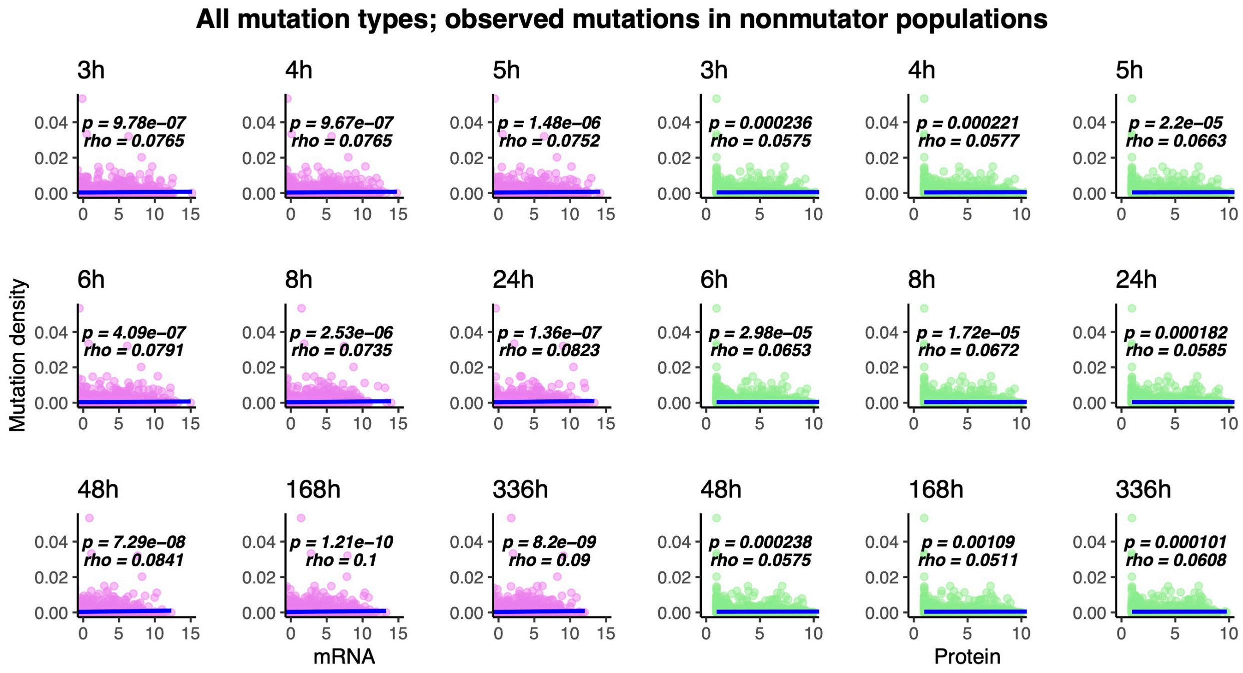

Supplementary Figure S5. The density of observed mutations per gene across all hypermutator LTEE populations at 60,000 generations anti-correlates with mRNA abundance in exponential phase for all 11 50,000 generation LTEE clones grown in DM4000 media. These transcriptomic data were reported by Favate et al. (2021). Statistically significant correlations are shown in blue, while non-significant correlations are shown in light gray. Spearman correlation coefficients (*rho*) and associated *p*-values are shown on each panel.

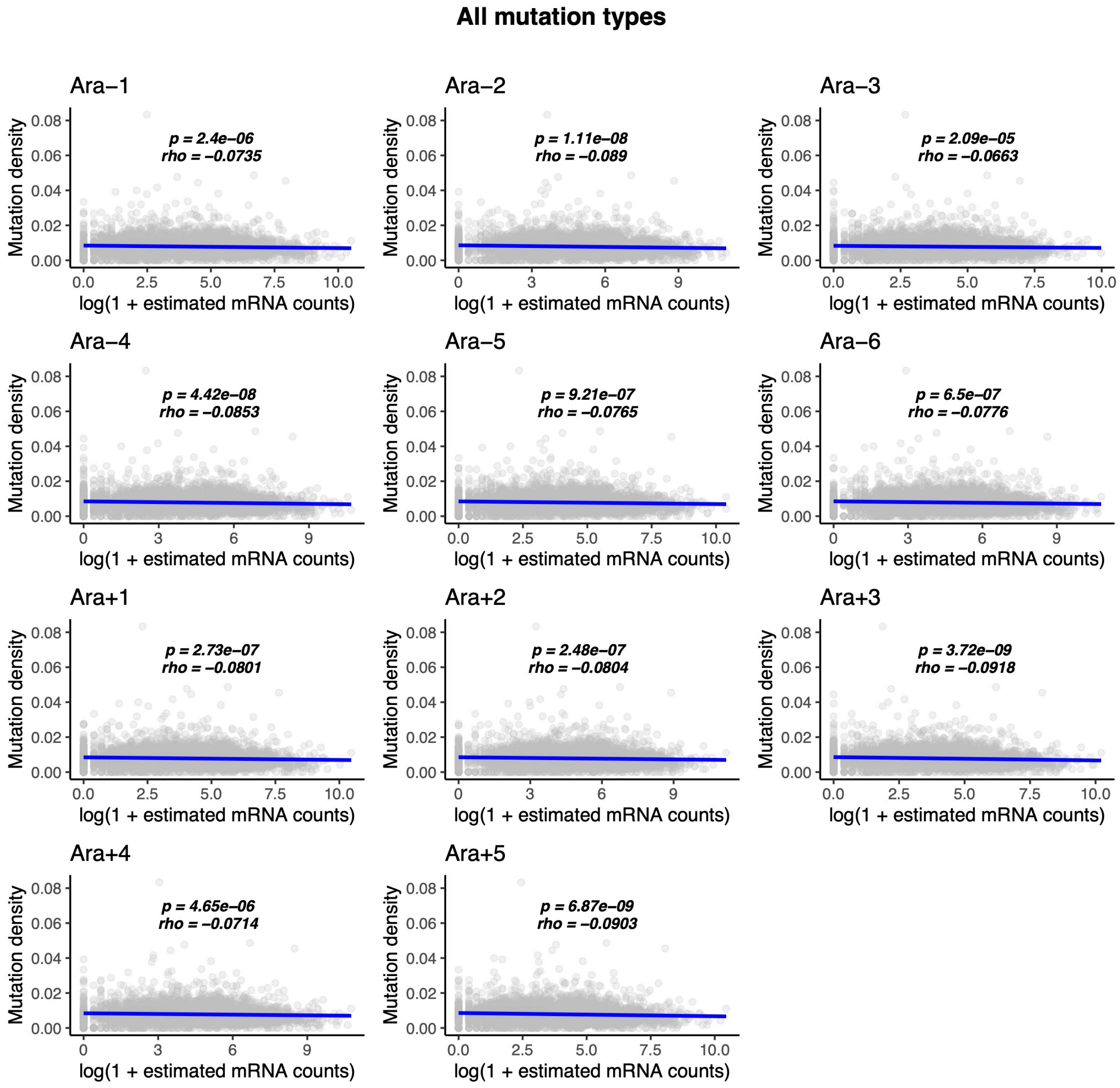

Supplementary Figure S6. The density of nonsynonymous mutations per gene across all hypermutator LTEE populations at 60,000 generations anti-correlates with mRNA abundance in exponential phase for all 11 50,000 generation LTEE clones grown in DM4000 media. These transcriptomic data were reported by Favate et al. (2021). See legend to Supplementary Figure S5 for more details.

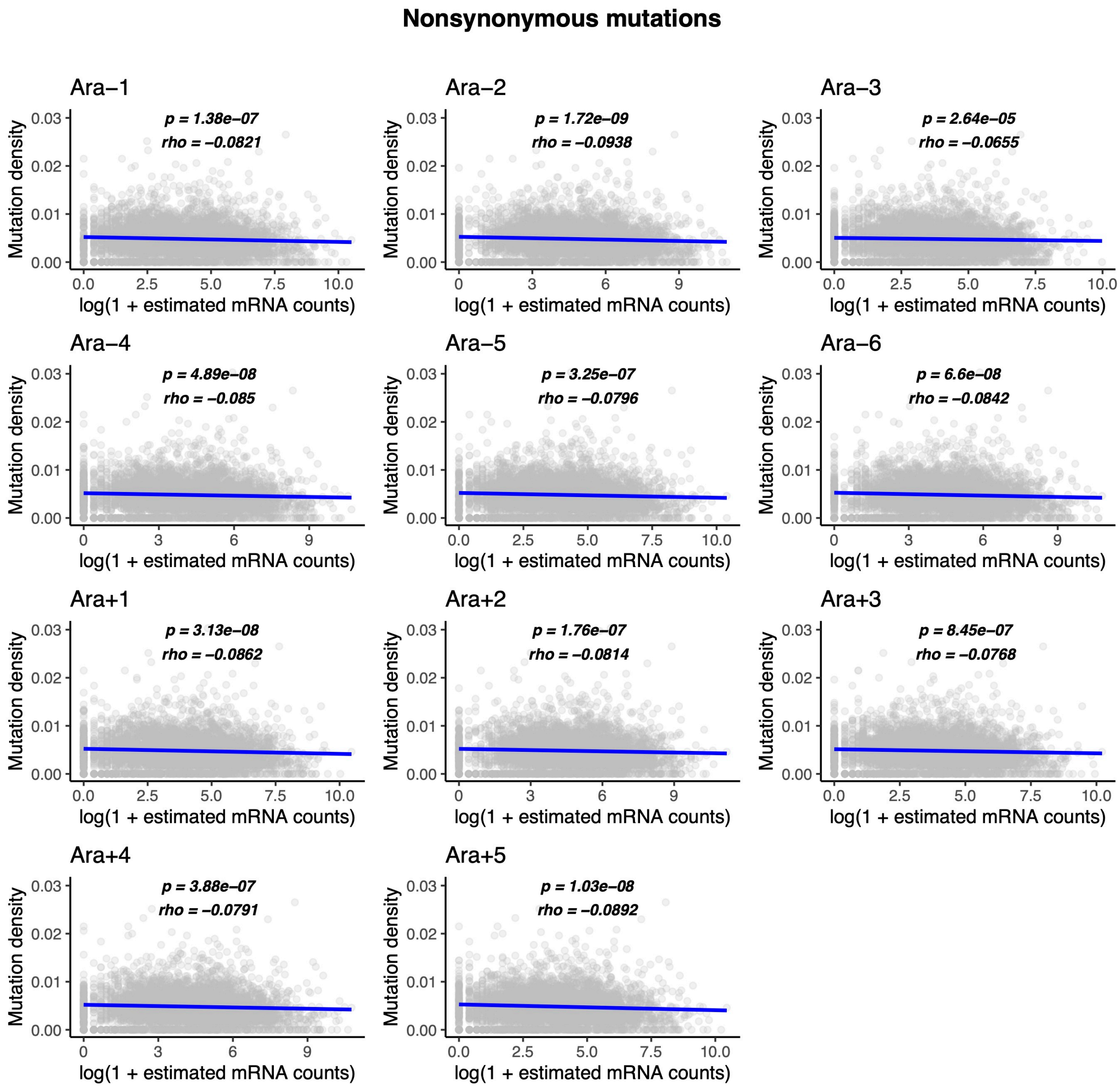

Supplementary Figure S7. The density of synonymous mutations per gene across all hypermutator LTEE populations at 60,000 generations positively correlates with mRNA abundance in exponential phase for all 11 50,000 generation LTEE clones grown in DM4000 media. These transcriptomic data were reported by Favate et al. (2021). See legend to Supplementary Figure S5 for more details.

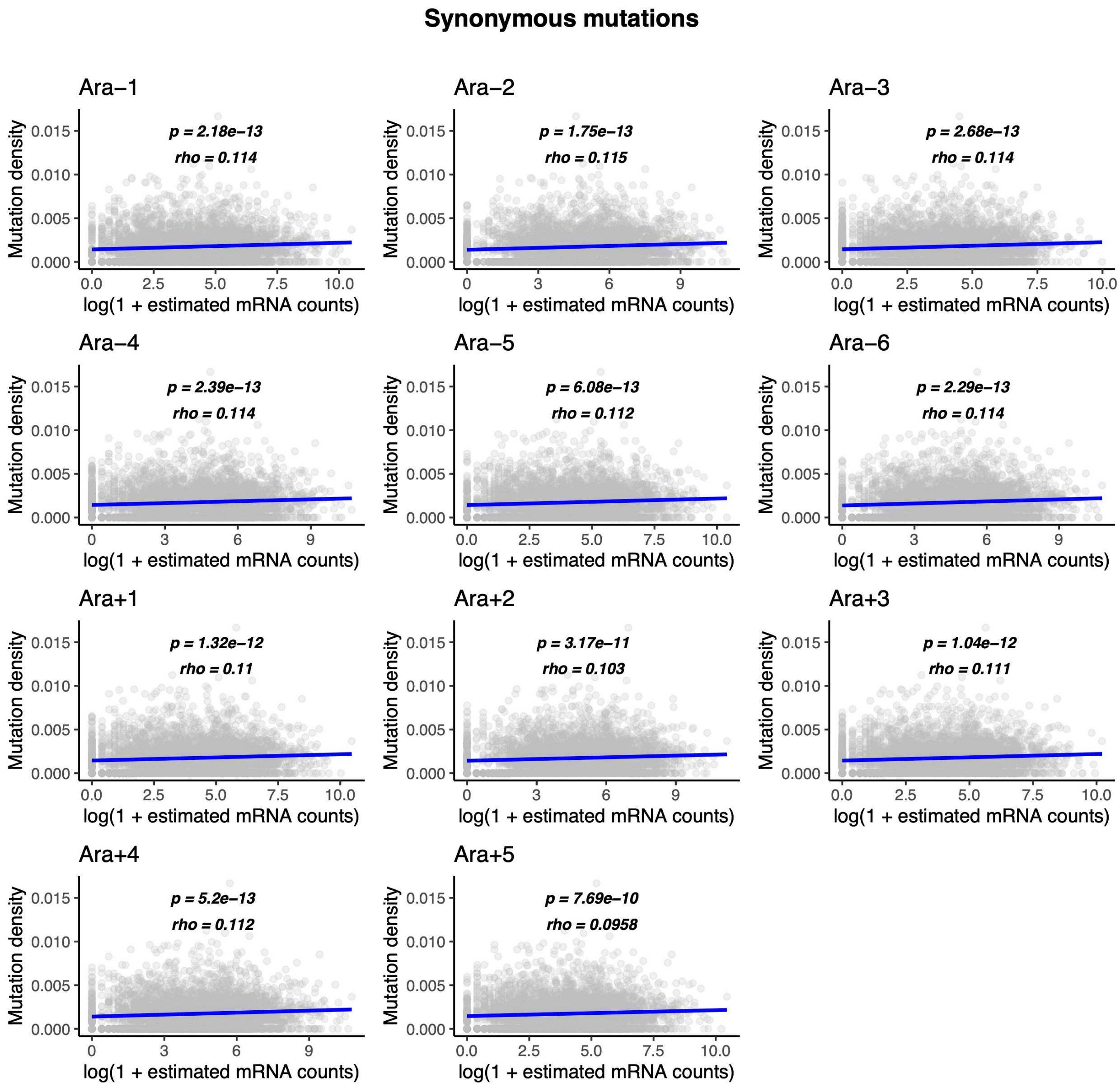

Supplementary Figure S8. The density of observed mutations per gene across all nonmutator LTEE populations at 60,000 generations positively correlates with mRNA abundance in exponential phase in most of the 11 50,000 generation LTEE clones grown in DM4000 media. These transcriptomic data were reported by Favate et al. (2021). See legend to Supplementary Figure S5 for more details.

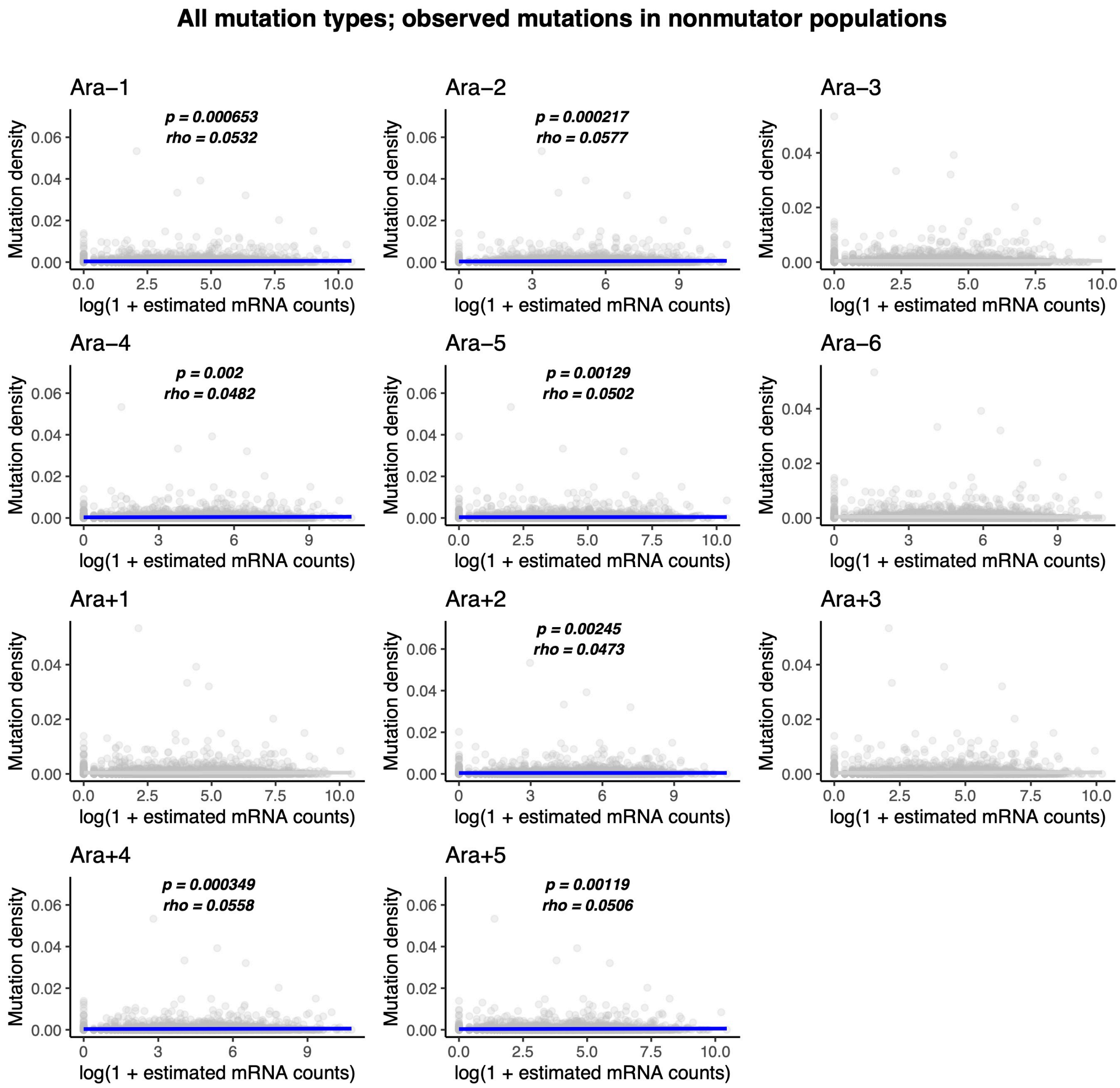

Supplementary Figure S9. The density of observed mutations per gene across all hypermutator LTEE populations at 60,000 generations anti-correlates with protein abundance data in the ProteomeVis database. The ProteomeVis database is described in Razban et al. (2018). Each point corresponds to one of 664 genes with abundance data in ProteomeViz. At the time of accession, ProteomeVis did not contain abundance data for the remaining 3541 genes in the 60,000 generations LTEE metagenomics data (Good et al. 2017). Corresponding results for synonymous mutations in the hypermutator LTEE populations, and for all mutation types in the nonmutator LTEE populations, are not statistically significant.

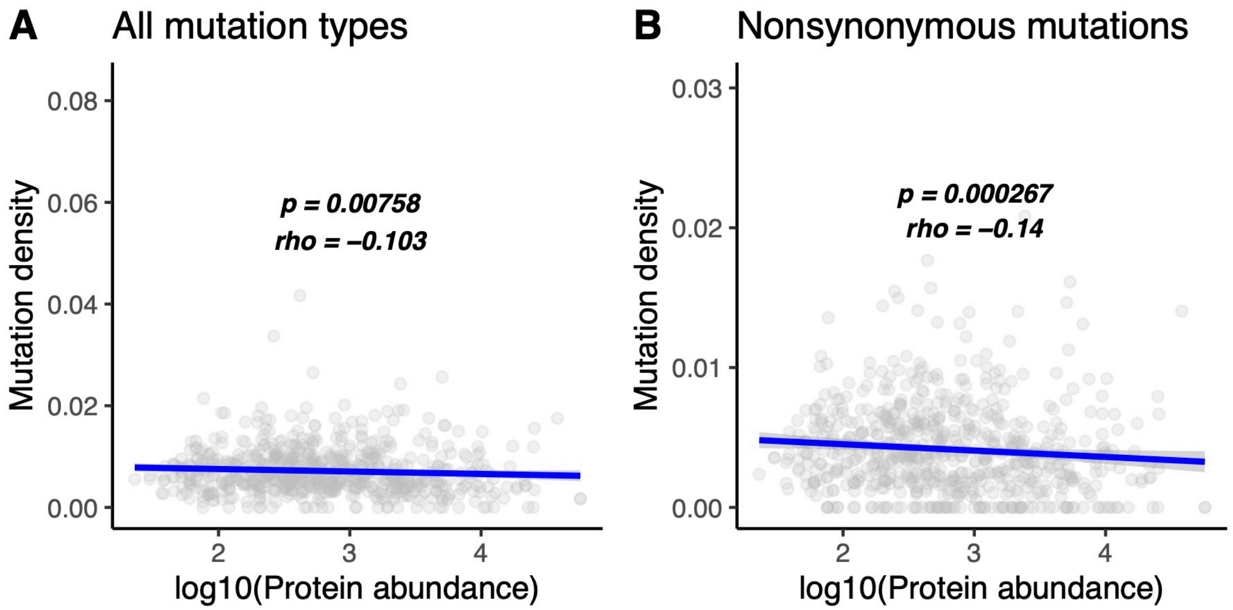

Supplementary Figure S10. The density of observed mutations per gene across all nonmutator LTEE populations does not correlate with PPI degree. A) Comparisons to the PPI data from Cong et al. (2019) are shown in light blue B) Comparisons to the PPI data from Zitnik et al. (2019) are shown in orange. C) Comparisons to the ProteomeVis PPI dataset are shown in red.

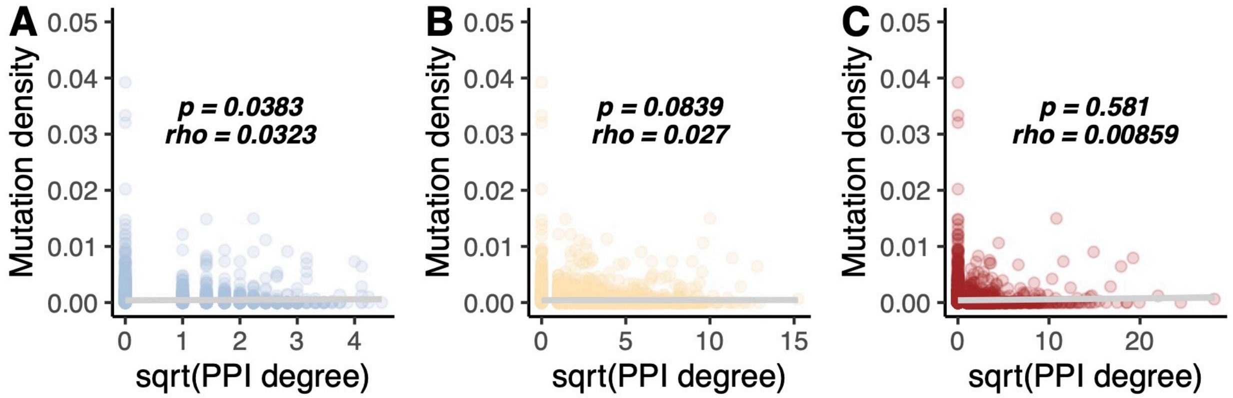

Supplement: evab070_Supplementary_Data [file evab070_supplementary_data.pdf]
